# 

**Published:** 1846-06

**Authors:** 


### CONTENTS

OF NO. VI.

---

### ORIGINAL COMMUNICATIONS.

### ESSAYS AND CASES.

ART. I.—An Essay on Diet in Health and Disease. By JOHN DAWSON, M.D., of Jamestown, Ohio, - - - - - - - - 461

ART. II.—Thoughts on Fever. By JAMES C. HARRIS, M.D., of Wetumpka, Alabama. - - - - - - - - - - - 483

ART. III.—Stricture of the Urethra, with Retention of the Urine; Division of the Stricture; Puncture of the Bladder; Recovery. By WILLIAM CALDWELL, M.D., of Louisville, Ky. - - - 494

ART. IV.—A Case of Pneumonia, succeeded by Purpura Hemorrhagica and Abscess. By W. H. STILWELL, M.D., of Madison county, Tennessee. - - - - - - - - - - - 500

### REVIEWS.

ART. V.—Homœopathy, Allopathy, and "Young Physic." By JOHN FORBES, M.D., F.R.S., &c., &c. - - - - - - - 506

ART. VI.—The Diagnosis, Pathology, and Treatment of the Diseases of the Chest. By W. W. GERHARD, M.D., Lecturer on Clinical Medicine to the University of Pennsylvania; one of the Physicians to the Pennsylvania Hospital, &c. Second edition, revised and enlarged. - - - - - - - - - - - - - 523

ART. VII.—A Compendium of the Lectures on the Theory and Practice of Medicine, delivered by Professor CHAPMAN in the University of Pennsylvania. Prepared with permission from Dr. Chapman's manuscripts, and published with permission. By N. D. BENEDICT, M.D., Fellow of the College of Physicians of Philadelphia; Physician to the Lying-in Department of the Philadelphia Hospital. - - - - - - - - - - - - - - - 523

ART. VIII.—Five Dissertations on Fever. By GEORGE FORDYCE, M. D., F.R.S., Fellow of the Royal College of Physicians; Senior Physician to St. Thomas's Hospital, and Reader on the Practice of Physic in London. 2d American edition, with an Introduction. 524

ART. IX.—A Clinical Introduction to the Practice of Auscultation, and other modes of Physical Diagnosis; intended to Simplify the Study of the Diseases of the Lungs and Heart. By H. M. HUGHES, M. D., Fellow of the Royal College of Physicians; Assistant Physician to Guy's Hospital, &c. - - - - - - - - - 525

ART. X.—The Practice of Surgery. By JAMES MILLER, F.R.S.E., F. R.C.S.E., Professor of Surgery in the University of Edinburgh; Surgeon to the Royal Infirmary; &c., &c., &c. - - - 526
ART. XI.—The Natural History and Diseases of the Human Teeth. By By JOSEPH FOX, M.R.C.S.L., Member of the Society of Medicine, Paris; &c., &c. First American, from the third London edition. Remodeled, with an introduction and numerous additions. By CHAPIN A. HARRIS, M.D., D.D.S., Professor of Practical Dentistry and Dental Pathology in the Baltimore College of Dental Surgeons, &c, &c. - . - - - - - - - - 527
ART. XII.—Elements of Physiology, including Physiological Anatomy, for the use of the Medical Student. By WM. B. CARPENTER, M.D., F.R.S., Fullerian Professor of Physiology in the Royal Institution of Great Britain, &c., &c. - - - - - - - 528
ART. XIII.—Elements of Chemistry, including the history of the Imponderables and the Inorganic Chemistry of the late EDWARD TURNER, M.D., F.R.S.L.&E. Seventh edition. And the Outlines of Organic Chemistry. By WM. GREGORY, M.D., &c., Professor of Chemistry in the University of Edinburgh. With Notes and Additions. By JAMES B. ROGERS, M.D., Professor of General Chemistry, Franklin Institute; &c., &c., and ROBERT E. ROGERS, M.D., &c., Professor of Chemistry and Materia Medica, University of Virginia, &c. - - - - - - - - - - 529
ART. XVI.—An Introduction to Entomology; or Elements of the Natural History of Insects. With Plates. By WM. KIRBY, M.A., F.R. S.&.L.S., Rector of Barham; and WM. SPENCE, Esq., F.R.S.& L.S. From the Sixth London edition, which was corrected and considerably enlarged. - - - - - - - - - - - 530

### SELECTIONS FROM AMERICAN AND FOREIGN JOURNALS.

Cause, Nature, and Treatment of Western Fevers. - - - - 533
On the Treatment of Placenta Prævia. - - - - - - - - 535
The Royal Academy of Medicine and the King of the French. - - 536
Rupture of the Stomach in a Boy. - - - - - - - - 537
Potato Disease. - - - - - - - - - - - - 539
Alleged Cure of Rheumatism. - - - - - - - - 539
Fever in Africa. - - - - - - - - - - - - 539
Devergie's Ointment for Chilblains. - - - - - - - - 540
Artificial Teeth. - - - - - - - - - - - - 540
Case of Extreme Diffusion of Odors. - - - - - - . 542
Opium Treatment of Rheumatism. - - - - - - - - 543
Premature Interments. - - - - - - - - - - 543
Asserted Injurious Effects of the Manufacture of Lucifer Matches. - 543
National Medical Convention. - - - - - - - - 544

### ORIGINAL INTELLIGENCE.

National Medical Convention. - - - - - - - - - 545
Asafœtida no Preventive of Measles. - - - - - - - - 548
A Case ol Hydrothorax successfully treated. - - - - - - 549
A Case of Self-Emasculation. - - - - - - - - - - 549

